# Increasing prevalence, time trend and seasonality of gastroschisis in São Paulo state, Brazil, 2005–2016

**DOI:** 10.1038/s41598-019-50935-1

**Published:** 2019-10-10

**Authors:** Mauricio Giusti Calderon, Edige Felipe de Sousa Santos, Luiz Carlos de Abreu, Rodrigo Daminello Raimundo

**Affiliations:** 1 Study Design and Scientific Writing Laboratory, Centro Universitário Saúde ABC, Av. Príncipe de Gales, 667. 2 floor, Santo André, 09060-870 SP Brazil; 2 Epidemiology Department, Faculdade de Saúde Pública da Universidade de São Paulo (USP), São Paulo, SP Brazil; 3 Public Policy and Local Development, Escola Superior de Ciências da Santa Casa de Misericórdia, Vitória, ES Brazil; 4 Graduate Entry Medical School, University of Limerick, Limerick, Ireland

**Keywords:** Epidemiology, Risk factors

## Abstract

To estimate the gastroschisis seasonality and trend of prevalence in recent years, stratified by maternal age and geographical clusters of São Paulo state, a population–based study was designed. We used data from the Live Births Information System (SINASC) in São Paulo state, Brazil, from 2005 to 2016. Trends of prevalence were evaluated for the specific subgroups using the Prais–Winsten regression model, and the Durbin–Watson test was used, to estimate the regression coefficient, the annual percent change (APC), and 95% confidence interval (CI). We observed 1576 cases of gastroschisis among 7,317,657 live births (LB), a prevalence of 2.154 (95% CI: 2.047–2.260) per 10,000 LB which included, 50.6% males, 67.4% Caucasians, 53.4% preterm births, and 80.9% caesarean births. The prevalence of gastroschisis significantly increased by 2.6% (95% CI: 0.0–5.2) per year, and this trend was higher in mothers aged 30–34 years (APC: 10.2, 95% CI: 1.4–19.4) than in mothers of other age groups. Between 2011 and 2016, we identified the existence of seasonality based on the date of conception in the middle months of the year (p = 0.002). This is the first and largest population–based study summarizing current epidemiology and identifying trend of prevalence of gastroschisis in São Paulo state.

## Introduction

Gastroschisis is the most common, abdominal wall defect in which paraumbilical herniation of gastrointestinal structures occurs into the amniotic cavity where it is not covered by any membrane^1–3^, although some authors consider it as an umbilical ring defect^4^. It is one of the main congenital diseases that require neonatal surgical intervention and is generally associated with high hospital costs, high neonatal morbidity, and mortality^1,5^. While risk factors for gastroschisis have been implicated, including maternal factors, dietary factors, and chemical exposures^6–8^, its etiology is still unknown. The prevalence of this defect has increased in recent decades^6,9–12^. According to Mastroiacovo *et al*., this increasing rate experienced worldwide is an epidemic^13^.

The aim of this study is to identify trends in prevalence, seasonality and risk factors of gastroschisis using São Paulo state population database.

## Results

From 2005–2016, 1576 gastroschisis cases were reported among 7,317,657 live births (LB) in São Paulo which included 50.6% males (95% CI: 48.1–53), 67.4% Caucasians (95% CI: 65.1–69.7), 53.4% preterm births (<37 weeks gestation), 64.5% births with low weight (<2500 g), 59.1% (95% CI: 56.6–61.5) with seven or more prenatal consultations, 98.4% (95% CI: 97.6–98.9) with single gestation, and 80.9% (95% CI: 78.9–82.8) caesarean births. According to the maternal age group, 691 (43.9%) were young mothers (<20 years) and 67.3% (95% CI: 64.9–69.5) of mothers were between 8 to 11 schooling years (Table 1).

**Table 1:** Maternal and Infant Sociodemographic characteristics (São Paulo state residents, 2005–2016).

Characteristics	Total Births (n = 7,318,152)	Gastroschisis cases (n = 1576)	Birth prevalence of gastroschisis per 10,000 births (95% CI)
**Infant Sex:**			
Male	3,748,988	797	2.13 (1.98: 2.27)
Female	3,568,165	761	2.13 (1.98: 2.28)
Ignored	999	18	180.18 (97.69: 262.67)
**Race:**			
White	4,811,694	1062	2.21 (2.07: 2.34)
Non–White	2,169,031	462	2.13 (1.94: 2.34)
Ignored	337,425	52	1.54 (1.12: 1.96)
**Gestational age (weeks):**			
22–27	34,410	20	5.81 (3.27: 8.36)
28–31	66,333	68	10.25 (7.82: 12.69)
32–36	602,713	753	12.49 (11.60: 13.39)
37–41	6,450,869	702	1.09 (1.01: 1.17)
>42	80,353	7	0.87 (0.23: 1.52)
Ignored	80,494	26	3.23 (1.99: 4.47)
**Birth weight (g):**			
>2500	6,627,484	555	0.84 (0.77: 0.91)
2499–1500	564,708	907	16.06 (15.02: 17.11)
<1500	103,601	109	10.52 (8.55: 12.50)
Ignored	22,359	5	2.24 (0.28: 4.20)
**Maternal Age (years):**			
<14	40,608	39	9.60 (6.59: 12.62)
15–19	1,070,311	652	6.09 (5.62: 6.56)
20–24	1,815,951	563	3.10 (2.84: 3.36)
25–29	1,878,813	201	1.07 (0.92: 1.22)
30–34	1,525,251	83	0.54 (0.43: 0.66)
35 +	986,540	38	0.39 (0.26: 0.51)
Ignored	678	—	—
**Maternal schooling (complete years):**			
0	23,784	1	0.42 (0: 1.24)
1–3	178,627	24	1.34 (0.81: 1.88)
4–7	1,258,372	331	2.63 (2.35: 2.91)
8–11	4,294,770	1060	2.47 (2.32: 2.62)
12 +	1,451,624	139	0.96 (0.80: 1.12)
Ignored	110,975	21	1.89 (1.08: 2.70)
**Multiple gestation**			
Singleton	7,318,152	1550	2.12 (2.01: 2.22)
Twin	162,737	25	1.54 (0.93: 2.14)
Triplet or higher	5,250	1	1.90 (0: 5.64)
Ignored	4,627	—	—
**Type of delivery:**			
Vaginal	3,030,744	301	0.99 (0.88: 1.11)
Caesarean section	4,280,677	1275	2.98 (2.82: 3.14)
Ignored	6,731	—	—
**Prenatal care consultations:**			
0	83,869	25	2.98 (1.81: 4.15)
1–3	277,724	122	4.39 (3.61: 5.17)
4–6	1,322,601	477	3.61 (3.28: 3.93)
7+	5,566,748	931	1.67 (1.57: 1.78)
Ignored	67,210	20	2.98 (1.67: 4.28)

**Figure 1:**
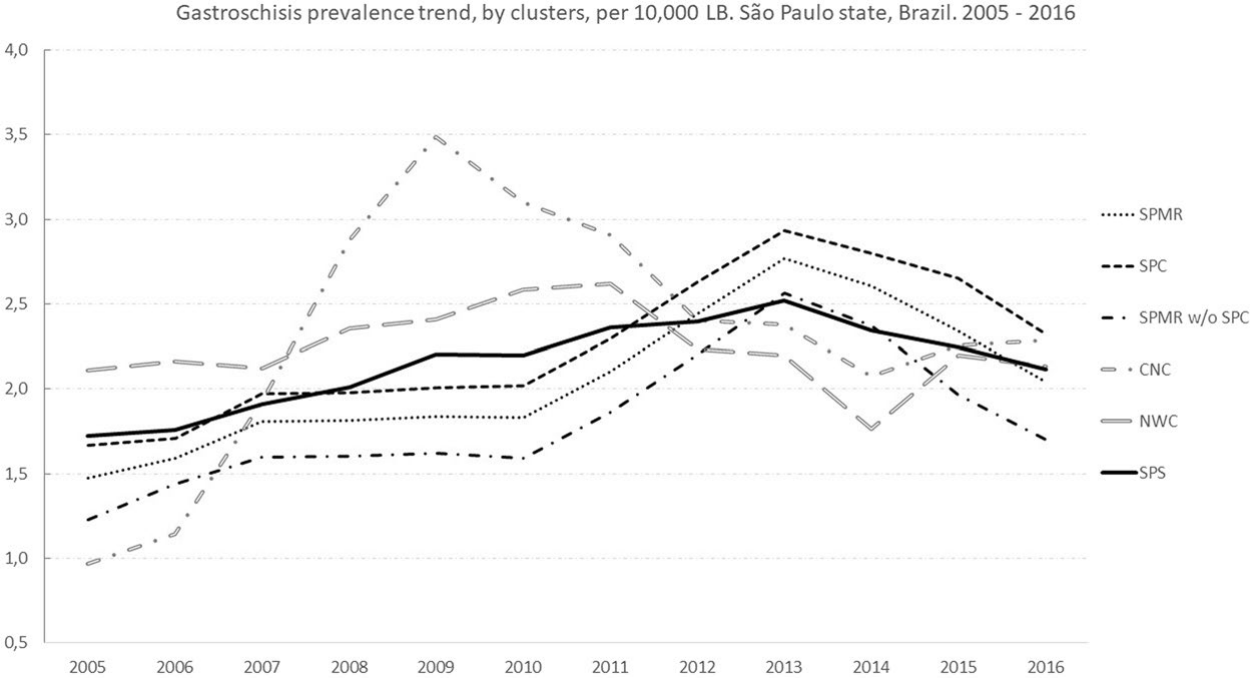
Gastroschisis prevalence trend, by cluster, per 10,000 LB. São Paulo State, Brazil, 2005–2016.

The rate of gastroschisis in São Paulo had increased from 1.75 cases per 10,000 LB in 2005 to 2.23 cases per 10,000 LB in 2016 (overall, 2.15 cases/10,000 LB; 95% CI: 2.05–2.26). The highest and lowest overall prevalence was in 2014 (2.54 cases/10,000 LB; 95% CI: 2.15–2.94) and in 2006 (1.71/10,000 LB; 95% CI: 1.38–2.04), respectively. The highest and lowest overall prevalence was in the Central-South cluster (2.68 cases/10,000 LB; 95% CI: 2.20–3.15) and Taubaté Administrative Region (1.79 cases/10,000 LB; 95% CI: 1.37–2.21), respectively. The highest and lowest number of cases was identified in São Paulo city (471) and Baixada Santista Metropolitan Region (66), respectively. In 2007, the Taubaté Administrative Region presented the lowest annual prevalence (0.31/10,000 LB; 95% CI: 0–0.92), and the highest annual prevalence was in the Central-South cluster (4.4/10,000 LB; 95% CI: 2.26–6.62) (Table 2). To facilitate the visualization of the trends in prevalence, in clusters that present significant results (p < 0.05), we reduced the random variation in the graph, using the third-order centred moving averages technique (Fig. 1).

**Table 2:** Number of cases, living births and prevalence of gastroschisis by clusters in São Paulo state, Brazil. 2005–2016 (per 10,000 Live Births).

Clusters	Years	2005	2006	2007	2008	2009	2010	2011	2012	2013	2014	2015	2016	Total
SPC	Cases	27	32	31	40	32	33	41	47	50	57	40	41	471
	LB	179,025	175,294	171,996	174,132	174,000	174,265	176,487	175,904	172,987	175,840	176,313	167,303	2,093,546
	Prevalence (95% CI)	1.51 (0.94: 2.08)	1.82 (1.19: 2.46)	1.80 (1.17: 2.43)	2.3 (1.58: 3.01)	1.84 (1.20: 2.47)	1.89 (1.25: 2.54)	2.32 (1.61: 3.03)	2.67 (1.91: 3.43)	2.89 (2.09: 3.69)	3.24 (2.40: 4.08)	2.27 (1.56: 2.97)	2.45 (1.70: 3.20)	2.25 (2.05: 2.45)
SPMR without SPC	Cases	16	18	25	22	18	26	21	30	41	37	23	24	301
	LB	140,552	135,985	134,772	135,975	134,921	135,764	138,073	139,999	139,133	142,475	145,968	138,782	1,662,399
	Prevalence (95% CI)	1.14 (0.58: 1.69)	1.32 (0.71: 1.93)	1.85 (1.13: 2.58)	1.62 (0.94: 2.29)	1.33 (0.72: 1.95)	1.91 (1.18: 2.65)	1.52 (0.87: 2.17)	2.14 (1.37: 2.91)	2.95 (2.04: 3.85)	2.6 (1.76: 3.43)	1.57 (0.93: 2.22)	1.73 (1.04: 2.42)	1.81 (1.60: 2.01)
SPMR	Cases	43	50	56	62	50	59	62	77	91	94	63	65	772
	LB	319,577	311,279	306,768	310,107	308,921	310,029	314,560	315,903	312,120	318,315	322,281	306,085	3,755,945
	Prevalence (95% CI)	1.34 (0.94: 1.75)	1.60 (1.16: 2.05)	1.82 (1.35: 2.30)	2.00 (1.50: 2.5)	1.62 (1.17: 2.07)	1.90 (1.42: 2.39)	1.97 (1.48: 2.46)	2.44 (1.89: 2.98)	2.91 (2.32: 3.51)	2.95 (2.35: 3.55)	1.95 (1.47: 2.44)	2.12 (1.61: 2.64)	2.05 (1.91: 2.20)
BSMR	Cases	6	7	5	6	2	7	7	4	5	6	7	4	66
	LB	25,555	24,874	25,304	25,157	24,227	24,360	25,159	25,773	24,978	25,373	25,287	23,925	299,972
	Prevalence (95% CI)	2.35 (0.47: 4.23)	2.81 (0.73: 4.9)	1.97 (0.24: 3.71)	2.38 (0.48: 4.29)	0.82 (0: 1.97)	2.87 (0.74: 5.00)	2.78 (0.72: 4.84)	1.55 (0.03: 3.07)	2.00 (0.25: 3.76)	2.36 (0.47: 4.26)	2.77 (0.72: 4.82)	1.67 (0.03: 3.31)	2.20 (1.67: 2.73)
TAR	Cases	6	7	1	7	4	7	6	12	8	3	4	6	71
	LB	33,247	32,681	32,005	32,502	32,153	32,762	33,331	33,245	33,166	34,190	34,187	32,893	396,362
	Prevalence (95% CI)	1.80 (0.36: 3.25)	2.14 (0.55: 3.73)	0.31 (0: 0.92)	2.15 (0.56: 3.75)	1.24 (0.02: 2.46)	2.14 (0.55: 3.72)	1.80 (0.36: 3.24)	3.61 (1.57: 5.65)	2.41 (0.74: 4.08)	0.88 (0: 1.87)	1.17 (0.02: 2.32)	1.82 (0.36: 3.28)	1.79 (1.37: 2.21)
CSC	Cases	13	5	8	8	12	16	7	13	10	10	10	8	120
	LB	39,132	37,765	36,690	36,392	35,617	36,025	36,708	37,633	37,474	38,236	39,286	37,366	448,324
	Prevalence (95% CI)	3.32 (1.51: 5.13)	1.32 (0.16: 2.48)	2.18 (0.67: 3.69)	2.2 (0.67: 3.72)	3.37 (1.46: 5.27)	4.44 (2.26: 6.62)	1.91 (0.49: 3.32)	3.45 (1.58: 5.33)	2.67 (1.01: 5.33)	2.61 (0.99: 4.23)	2.54 (0.97: 4.12)	2.14 (0.66: 3.62)	2.68 (2.2: 3.15)
CRC	Cases	21	18	18	17	15	23	23	13	22	18	18	23	229
	LB	83,263	82,435	81,184	83,124	83,410	84,395	84,949	87,225	87,371	89,480	91,880	87,167	1,025,883
	Prevalence (95% CI)	2.52 (1.44: 3.60)	2.18 (1.17: 3.19)	2.22 (1.19: 3.24)	2.04 (1.07: 3.02)	1.8 (0.89: 2.71)	2.72 (1.61: 3.84)	2.71 (1.60: 3.81)	1.49 (0.68: 2.30)	2.52 (1.46: 3.57)	2.01 (1.08: 2.94)	1.96 (1.05: 2.86)	2.64 (1.56: 3.72)	2.23 (1.94: 2.52)
CNC	Cases	7	5	9	21	22	20	14	19	11	14	14	14	170
	LB	62,526	60,909	60,413	60,411	59,903	60,208	60,693	61,269	60,827	62,931	63,666	59,733	733,489
	Prevalence (95% CI)	1.12 (0.29: 1.95)	0.82 (0.10: 1.54)	1.49 (0.52: 2.46)	3.47 (1.99: 4.96)	3.67 (2.14: 5.21)	3.32 (1.86: 4.78)	2.31 (1.1: 3.51)	3.10 (1.71: 4.49)	1.81 (0.74: 2.87)	2.22 (1.06: 3.39)	2.2 (1.04: 3.35)	2.34 (1.11: 3.57)	2.32 (1.97: 2.66)
NWC	Cases	12	11	12	11	15	13	14	16	7	14	9	14	148
	LB	55,386	53,401	52,848	54,097	54,236	53,566	54,810	55,548	54,945	57,147	57,430	54,252	657,666
	Prevalence (95% CI)	2.17 (0.94: 3.39)	2.06 (0.84: 3.28)	2.27 (0.98: 3.55)	2.03 (0.83: 3.23)	2.76 (1.36: 4.16)	2.43 (1.11: 3.74)	2.55 (1.21: 3.89)	2.88 (1.47: 4.29)	1.27 (0.33: 2.22)	2.45 (1.17: 3.73)	1.57 (0.54: 2.59)	2.58 (1.23: 3.93)	2.25 (1.89: 2.61)
SPS	Cases	108	103	109	132	120	145	133	154	154	159	125	134	1576
	LB	618,686	603,344	595,212	601,790	598,467	601,345	610,210	616,596	610,881	625,672	634,017	601,437	7,317,657
	Prevalence (95% CI)	1.74 (1.42: 2.08)	1.71 (1.38: 2.04)	1.83 (1.49: 2.18)	2.19 (1.82: 2.57)	2.00 (1.65: 2.36)	2.41 (2.02: 2.80)	2.18 (1.81: 2.55)	2.5 (2.10: 2.89)	2.52 (2.12: 2.92)	2.54 (2.15: 2.94)	1.97 (1.63: 2.32)	2.23 (1.85: 2.61)	2.15 (2.05: 2.26)

Source: Live Births Information System (SINASC). Data: Unified Health System Department of Informatics (DATASUS – www.datasus.saude.gov.br). Ministry of Health. Brazil.

During 2005–2016, São Paulo state, São Paulo Metropolitan Region and São Paulo City presented an increasing trend of prevalence of gastroschisis, with an annual percent change rate of 2.6%, 4.7%, and 5.0% respectively. According to 2 periods of 6 years each (2005–2010 and 2011–2016), São Paulo state presents an increasing trend of prevalence of gastroschisis only in the first period (APC = 6.7%; 95% CI: 4.0–9.6). Moreover, similar results were found at the Central–North cluster (APC = 36.1%; 95% CI: 8.4–71.4) and at the Northwest cluster (APC = 4.7%; 95% CI: 0.9–8.6) in the second period. All other clusters presented stationary trends (Table 3).

**Table 3:** Prais–Winsten regression model for trends in gastroschisis prevalence, by clusters in São Paulo state, Brazil. 2005–2016.

Clusters	β (CI 95%) 2005–2010	*APC %* (CI 95%) *2005–2010*	Reading	β (CI 95%) 2011–2016	*APC %* (CI 95%) *2011–2016*	Reading	β (CI 95%) 2005–2016	*APC %* (CI 95%) *2005*–*2016*	Reading
SPC	0.017 (−0.016: 0.051)	4.0 (−3.6: 12.5)	Stationary	−0.001 (−0.048: 0.046)	−0.2 (−10.5: 11.2)	Stationary	0.021 (0.007: 0.035)	5.0 (1.6: 8.4)	Increasing
SPMR without SPC	0.030 (−0.019: 0.079)	7.2 (−4.3: 19.9)	Stationary	0.001 (−0.103: 0.105)	0.2 (−21.1: 27.4)	Stationary	0.019 (−0.005: 0.042)	4.5 (−1.1: 10.2)	Stationary
SPMR	0.023 (−0.012: 0.059)	5.4 (−2.7: 14.6)	Stationary	0.000 (−0.069: 0.068)	0 (−14.7: 16.9)	Stationary	0.020 (0.001: 0.038)	4.7 (0.2: 9.1)	Increasing
BSMR	−0.050 (−0.137: 0.037)	−10.9 (−27.1: 8.9)	Stationary	−0.003 (−0.075: 0.069)	−0.7 (−15.9: 17.2)	Stationary	−0.001 (−0.025: 0.023)	−0.3 (−5.6: 5.3)	Stationary
TAR	0.014 (−0.164: 0.192)	3.3 (−31.5: 55.6)	Stationary	−0.054 (−0.197: 0.088)	−11.7 (−36.5: 22.5)	Stationary	0.009 (−0.037: 0.055)	2.1 (−8.1: 13.4)	Stationary
CSC	0.053 (−0.063: 0.170)	13.0 (−13.5: 47.9)	Stationary	−0.006 (−0.066: 0.054)	−1.4 (−14.1: 13.2)	Stationary	0.005 (−0.020: 0.030)	1.2 (−4.4: 7.0)	Stationary
CRC	−0.003 (−0.051: 0.044)	−0.7 (−11.1: 10.7)	Stationary	0.009 (−0.035: 0.053)	2.1 (−7.7: 13.0)	Stationary	−0.001 (−0.013: 0.010)	−0.3 (−3.0: 2.4)	Stationary
CNC	0.134 (0.035: 0.234)	36.1 (8.4: 71.4)	Increasing	−0.014 (−0.050: 0.022)	−3.2 (−10.9: 5.2)	Stationary	0.026 (−0.026: 0.078)	6.2 (−5.8: 19.7)	Stationary
NWC	0.020 (0.004: 0.036)	4.7 (0.9: 8.6)	Increasing	−0.020 (−0.085: 0.045)	−4.5 (−17.8: 10.9)	Stationary	−0.005 (.0.019: 0.009)	−1.1 (−4.3: 2.1)	Stationary
SPS	0.028 (0.017: 0.04)	6.7 (4.0: 9.6)	Increasing	−0.008 (−0.038: 0.023)	−1.8 (−8.4: 5.4)	Stationary	0.011 (0.000: 0.022)	2.6 (0.0: 5.2)	Increasing

β –regression coefficient, 95% CI – confidence interval 95%, APC = annual percent change.

Source: Live Births Information System (SINASC). Data: Unified Health System Department of Informatics (DATASUS – www.datasus.saude.gov.br). Ministry of Health. Brazil.

**Figure 2:**
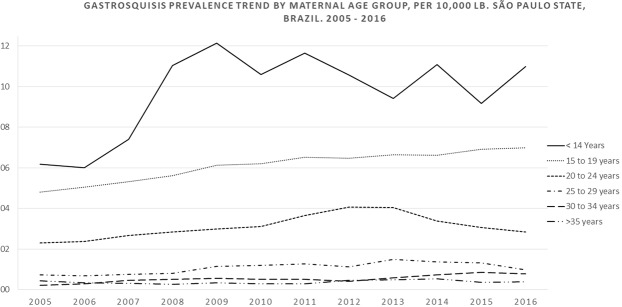
Gastroschisis prevalence trend, by maternal age group, per 10,000 LB. São Paulo State, Brazil, 2005–2016.

In young mothers (<20 years), the number of LB decreased by 24.4%, from 2005 (104,919) to 2016 (79,298), whereas in older mothers (≥30 years), there was an increase of 30% (182,438 to 237,213), in the same period. Moreover, 691 (43.84%) and 121 (7.68%) cases of gastroschisis were detected in young mothers and older mothers, respectively. The highest gastroschisis prevalence was in mothers aged ≤14 years (9.6/10,000 LB; 95% CI: 6.59–12.62) followed by 15–19 years (6.09/10,000 LB; 95% CI: 5.62–6.56), 20–24 years (3.1/10,000 LB; 95% CI: 2.84–3.36), 25–29 years (1.07/10,000 LB; 95% CI: 0.92–1.22), 30–34 years (0.54/10,000 LB; 95% CI: 0.54–0.66) and ≥35 years (0.39/10,000 LB; 95% CI: 0.26–0.51) (Table 4). Further, to facilitate the visualization of the gastroschisis trends in prevalence by maternal age group, we used the third-order centred moving averages technique (Fig. 2).

**Table 4:** Number of cases, living births and prevalence of gastroschisis by maternal age in São Paulo state, Brazil. 2005–2016 (per 10,000 Live Births).

Maternal Age (years):	year	2005	2006	2007	2008	2009	2010	2011	2012	2013	2014	2015	2016	Total
≤14	Cases	3	1	2	5	5	3	3	6	2	2	7	—	39
	LB	3,163	3,458	3,536	3,652	3,636	3,347	3,315	3,547	3,472	3,604	3,187	2,691	40,608
	Prevalence (95% CI)	9.48 (0: 20.21)	2.89 (0: 8.56)	5.66 (0: 13.49)	13.69 (1.7: 25.68)	13.75 (1.71: 25.8)	8.96 (0: 19.1)	9.05 (0: 19.29)	16.92 (3.39: 30.44)	5.76 (0: 13.74)	5.55 (0: 13.24)	21.96 (5.71: 38.22)	—	9.60 (6.59: 12.62)
15–19	Cases	53	43	52	55	47	61	54	55	61	59	52	60	652
	LB	101,756	97,632	93,815	91,146	89,175	85,704	86,810	87,978	87,744	87,481	84,463	76,607	1,070,311
	Prevalence (95% CI)	5.21 (3.81: 6.61)	4.40 (3.09: 5.72)	5.54 (4.04: 7.05)	6.03 (4.44: 7.63)	5.27 (3.76: 6.78)	7.12 (5.33: 8.90)	6.22 (4.56: 7.88)	6.25 (4.60: 7.90)	6.95 (5.21: 8.70)	6.74 (5.02: 8.46)	6.16 (4.48: 7.83)	7.83 (5.85: 9.81)	6.09 (5.62: 6.56)
20–24	Cases	39	38	40	49	44	45	53	65	60	50	35	45	563
	LB	171,531	163,226	157,459	154,904	154,289	151,830	149,516	147,094	141,658	142,751	144,427	137,266	1,815,951
	Prevalence (95% CI)	2.27 (1.56: 2.99)	2.33 (1.59: 3.07)	2.54 (1.75: 3.33)	3.16 (2.28: 4.05)	2.85 (2.01: 3.69)	2.96 (2.10: 3.83)	3.54 (2.59: 4.50)	4.42 (3.34: 5.49)	4.24 (3.16: 5.31)	3.50 (2.53: 4.47)	2.42 (1.62: 3.23)	3.28 (2.32: 4.24)	3.10 (2.84: 3.36)
25–29	Cases	8	15	9	12	17	26	14	20	19	31	14	16	201
	LB	159,738	156,205	156,885	159,997	157,330	158,644	158,696	157,462	153,147	156,029	157,036	147,644	1,878,813
	Prevalence (95% CI)	0.50 (0.15: 0.85)	0.96 (0.47: 1.45)	0.57 (0.20: 0.95)	0.75 (0.33: 1.17)	1.08 (0: 1.59)	1.64 (1.01: 2.27)	0.88 (0.42: 1.34)	1.27 (0.71: 1.83)	1.24 (0.68: 1.80)	1.99 (1.29: 2.69)	0.89 (0.42: 1.36)	1.08 (0.55: 1.61)	1.07 (0.92: 1.22)
30–34	Cases	2	3	5	8	5	7	7	6	4	14	13	9	83
	LB	112,972	112,151	112,497	117,888	118,459	123,965	129,935	135,359	137,825	141,715	144,949	137,536	1,525,251
	Prevalence (95% CI)	0.18 (0: 0.42)	0.27 (0: 0.57)	0.44 (0: 0.83)	0.68 (0.21: 1.15)	0.42 (0.05: 0.79)	0.56 (0.15: 0.98)	0.54 (0.14: 0.94)	0.44 (0.09: 0.80)	0.29 (0.01: 0.57)	0.99 (0: 1.51)	0.90 (0.41: 1.38)	0.65 (0.23: 1.08)	0.54 (0.43: 0.66)
≥35	Cases	3	3	1	3	2	3	2	2	8	3	4	4	38
	LB	69,466	70,666	70,943	74,192	75,580	77,848	81,931	85,153	87,038	94,097	99,949	99,677	986,540
	Prevalence (95% CI)	0.43 (0: 0.92)	0.42 (0: 0.90)	0.14 (0: 0.42)	0.40 (0: 0.86)	0.26 (0: 0.63)	0.39 (0: 0.82)	0.24 (0: 0.58)	0.23 (0: 0.56)	0.92 (0.28: 1.56)	0.32 (0: 0.68)	0.40 (0.01: 0.79)	0.40 (0.01: 0.79)	0.39 (0.26: 0.51)
Total	Cases	108	103	109	132	120	145	133	154	154	159	125	134	1576
	LB	618,686	603,344	595,212	601,790	598,467	601,345	610,210	616,596	610,881	625,672	634,017	601,437	7,317,657
	Prevalence (95% CI)	1.74 (1.42: 2.08)	1.71 (1.38: 2.04)	1.83 (1.49: 2.17)	2.19 (1.82: 2.57)	2.00 (1.64: 2.36)	2.41 (2.02: 2.80)	2.18 (1.81: 2.55)	2.5 (2.10: 2.89)	2.52 (2.12: 2.92)	2.54 (2.14: 2.93)	1.97 (1.62: 2.32)	2.23 (1.85: 2.60)	2.15 (2.05: 2.26)

Source: Live Births Information System (SINASC). Data: Unified Health System Department of Informatics (DATASUS – www.datasus.saude.gov.br). Ministry of Health. Brazil.

The overall prevalence of gastroschisis increased over the course of the study period (2005–2016) in three out of five maternal age groups (Table 4, Fig. 2). The highest increase in gastroschisis prevalence occurred in mothers aged 30–34 years (APC = 10.2%; 95% CI: 1.4–19.4) followed by mothers aged 25–29 years (APC = 6.9%; 95% CI: 0.9–13.0) and 15–19 years (APC = 3.5%; 95% CI: 2.1–5.0), the other age groups presented a stationary trend. A significant increase in gastroschisis prevalence occurred in the 20–24-year age group until 2012, followed by a progressive decrease until 2015, and in 2016, a new increase in the peak was observed that lead to an overall stationary result. In the first period (2005–2010), the highest increasing trend of prevalence occurred in the maternal age group of 25–29 years (APC = 19.7% 95% CI: 0.7 to 42.2). However, in the second period (2011–2016), all maternal age groups had a stationary prevalence trend. (Table 5)

**Table 5:** Prais–Winsten regression model for trends in gastroschisis prevalence, by maternal age in São Paulo state, Brazil. 2005–2016.

Maternal Age (years):	β (CI 95%) 2005–2010	*APC %* (CI 95%) *2005–2010*	Reading	β (CI 95%) 2011–2016	*APC %* (CI 95%) *2011–2016*	Reading	β (CI 95%) 2005–2016	*APC %* (CI 95%) *2005–2016*	Reading
≤14	0.09 (−0.090: 0.232)	23.2 (−18.8: 70.4)	Stationary	0.020 (−0.270: 0.309)	4.6 (−46.3:103.8)	Stationary	0.027 (−0.03: 0.08)	6.42 (−6.7: 20.2)	Stationary
15–19	0.027 (0.005: 0.049)	6.4 (1.2: 12.0)	Increasing	0.012 (−0.007: 0.030)	0.27 (−1.5: 7.1)	Stationary	0.015 (0.009: 0.021)	3.5 (2.1: 5.0)	Increasing
20–24	0.027 (0.006: 0.048)	6.4 (1.4: 11.7)	Increasing	−0.032 (−0.084: 0.020)	−7.1 (−17.6: 4.7)	Stationary	0.015 (−0.007: 0.036)	3.5 (−1.6: 8.6)	Stationary
25–29	0.078 (0.003: 0.153)	19.7 (0.7: 42.2)	Increasing	0.004 (−0.083: 0.091)	0.9 (−17.4: 23.3)	Stationary	0.029 (0.004: 0.053)	6.9 (0.9: 13.0)	Increasing
30–34	0.091 (−0.007: 0.198)	23.3 (−1.6: 57.8)	Stationary	0.060 (−0.52: 0.172)	14.8 (−11.3: 48.6)	Stationary	0.042 (0.006: 0.077)	10.2 (1.4: 19.4)	Increasing
≥35	−0.012 (−0.116: 0.092)	−2.7 (−23.4: 23.6)	Stationary	0.036 (−0.084: 0.155)	8.6 (−17.6: 42.9)	Stationary	0.014 (−0.014: 0.041)	3.3 (−3.2: 9.9)	Stationary

β –regression coefficient, 95% CI – confidence interval 95%, APC = annual percent change.

Source: Live Births Information System (SINASC). Data: Unified Health System Department of Informatics (DATASUS – www.datasus.saude.gov.br). Ministry of Health. Brazil.

**Figure 3:**
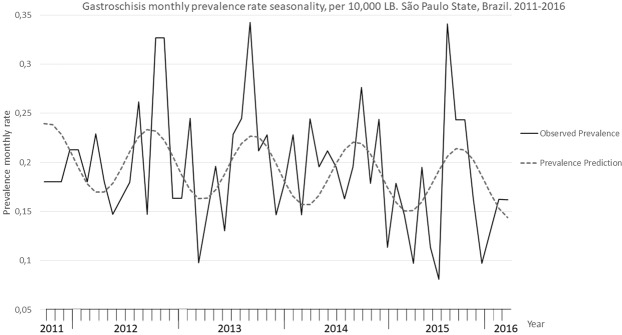
Gastroschisis prevalence seasonality. São Paulo state, Brazil, 2011–2016.

In 2011–2016 a significant seasonal variation in gastrosquisis monthly prevalence rate, based on the month of conception, was found, and it was, on average, 7.4% higher in the middle months of the year compared to the end or beginning months (95% CI: 0.013–0.053; p = 0.002) (Fig. 3).

## Discussion

To the best of our knowledge, this is the first population–based study that used the Live Births Information System in São Paulo state to describe the recent seasonality, and prevalence trend of gastroschisis in Brazil.

The prevalence of gastroschisis in São Paulo state from 2005–2016 was 2.15 (95% CI: 2.05–2.26) cases per 10,000 LB, and it significantly increased by 27.61% from 1.74 (95% CI: 1.42–2.08) to 2.23 (95% CI: 1.85–2.61) cases per 10,000 LB, with an increasing annual percent change of 2.6% (95% CI: 0.0–5.2). The increasing prevalence in São Paulo state is concordant with other studies worldwide^3,9–11,14–18^, but not with Li *et al*.^2^, which found a decreasing prevalence of gastroschisis in all 14 cities of the Liaoning Province in China from 6.87 to 0.80 per 10,000 LB during 2006–2015.

Although an increasing trend in gastroschisis prevalence was observed in São Paulo from 2005–2016 (APC = 2.6%; 95% CI: 0.0–5.2), significant increasing trend results were observed only in São Paulo city (APC = 5.0%; 95% CI: 1.6–8.4) and São Paulo Metropolitan Region (APC = 4.7%; 95% CI: 0.2–9.1), and this is because all gastroschisis cases in São Paulo city were counted in the São Paulo Metropolitan region as well. When we excluded these cases from the São Paulo Metropolitan Region (SPMR without SPC), the result is a stationary trend (APC = 4,5%; 95% CI: –0.1–10.2) similar to other presented clusters.

In the first period (2005–2010), São Paulo state presented a significant increasing trend in gastroschisis prevalence (APC = 6.7%; 95% CI: 4.0–9.6) and again, only two clusters presented a significant increasing trend, the Central North cluster (APC = 36.1%; 95% CI: 8.4–71.4) and the Northwest cluster (APC = 4.7%; 95% CI: 0.9–8.6), and all other clusters presented a stationary trend. Considering that the Central North cluster and Northwest cluster are predominantly rural areas, it can be suggested that, as in other studies^6–8^, that agricultural exposures, such as the use of pesticides, may have influenced the increasing number of gastroschisis cases in these clusters. At the second period (2011–2016), São Paulo state and all clusters presented a stationary trend, and this is mainly explained by the decrease in gastroschisis prevalence observed since 2015.

The association between young maternal age and gastroschisis is a well–documented risk factor^14–16,19,20^. However, the increase in gastroschisis prevalence is not because of an increase in teen births, since birth rates have decreased among mothers <25 years, and the highest increase in prevalence trend occurred among mothers aged 30–34 years. Our results confirm that young maternal age is a significant risk factor for gastroschisis in São Paulo state. The highest gastroschisis prevalence was in the maternal age group <14 years (9.6 cases/10,000 LB; 95% CI: 6.59–12.62). The lowest gastroschisis prevalence was in the maternal age group >35 years (0.39 cases/10,000 LB; 95% CI: 0.26–0.51). Moreover, the number of LB decreased by 19,76% in the young maternal age groups such as, <14 years, 15–19 years, 20–24 years and 25–29 years, from 2005 (436,188 LB) to 2016 (364,208 LB), and in the same period, the number of gastroschisis cases increased by 17.47%, from 103 (2005) to 121 (2016).

The highest increase in gastroschisis trend of prevalence from 2005–2016 occurred in the maternal age group 30–34 years (APC = 10.2%; 95% CI: 1.4–19.4) despite having a stationary trend in the separated periods (2005–2010 and 2011–2016). In the first period (2005–2010), the highest increase in gastroschisis trend of prevalence occurred among the maternal age group 25–29 years (APC = 19.7%; 95% CI: 0.7–42.2), and at the second period (2011–2016) all maternal age groups presented a stationary trend.

Our findings are consistent with those of other studies that showed that neonates born with gastroschisis are often Caucasian, born preterm, and with low birthweight^8,14,15,21,22^. Most mothers usually have low educational status, and they are more likely to give birth by cesarean delivery, as in USA^1,10,23^ and Australia^18,24^. In the Kirollos *et al*.^25^ meta–analysis report, no quantifiable advantage of caesarean section over a vaginal delivery was observed for infants with gastroschisis. In the Salihu *et al*.^26^ study, the mode of delivery was not found to be associated with neonatal survival of infants with gastroschisis. Skarsgard^27^ suggest that as caesarean delivery has not demonstrated any benefits, vaginal delivery should be preferred, unless obstetric factors dictate otherwise.

We observed a significant increase in gastroschisis prevalence when conceptions occurred during the winter months (June, July and August) from May 2011 to April 2016 (95% CI 0.01–0.05, p = 0.002). Waller *et al*.^8^ found that from 1987 to 2006 in the Washington state, 805 cases of gastroschisis were detected in conceptions during the spring period. However, from 1995 to 2012 in California state, Anderson *et al*.^10^ reported that the risk of gastroschisis did not vary by the season of conception.

This is the first study to demonstrate a significant seasonal variation in the most important state of a large middle–income country such as Brazil, and this may raise some questions: Can the influence of climate change, or ambient air pollution, the use of vaccines or drugs before and/or during pregnancy influence the occurrence of gastroschisis?

There are some limitations to this study. There may have been mistakes during the registration of designations for congenital anomalies in the live birth declaration at the public database. Moreover, we did do not have data regarding stillbirths with gastroschisis.

The strength of this study are as follows. It is a population–based study describing the time trend and seasonality of gastroschisis prevalence in the most populous state in Brazil^28^ with the highest birth rate^28^ and with reliable public database^29,30^. We studied a relatively long time period of data up to 2016 and used, the most recent reports from the Brazilian Unified Health System (DATASUS), which allowed us to analyse both in the total period and in the 2 subperiods of 6 years each.

This is the first and largest population–based study summarizing the current epidemiology and identifying trend of prevalence and seasonality of gastroschisis in the São Paulo state. Our findings demonstrate an increasing trend of prevalence in São Paulo state in recent years, being higher in older mothers and in São Paulo city. The highest overall prevalence was in the Central–South cluster, and the lowest was in Taubaté administrative region. Significant seasonal variation of gastroschisis prevalence was found, being higher, when conceptions occurred during the winter months of the year during 2011–2016.

## Methods

### Study design & settings

This is a population–based study with time trend^31^, using official microdata of all cases of gastroschisis identified by the Live Births Information System (SINASC – Sistema de Informação sobre Nascidos Vivos) in São Paulo state, Brazil, from 2005 to 2016 and using data from the Unified Health System Department of Informatics^32^ (DATASUS – Departamento de Informática do Sistema Único de Saúde), maintained by the Ministry of Health of Brazil. We used the International Classification of Diseases 10^th^ Edition^33^, code Q79.3 to identify all the cases of gastroschisis, at the LB declaration, among all LB in the period of study.

The unit of analysis selected for this study was São Paulo state, which is the most populous state in Brazil, with a population (41,262,199 in 2010)^28^ and birth rate (610,000/year)^32^ comparable to many countries in Europe^34–36^ and Latin America^37,38^, and where the completeness of public data is more reliable^29,30^ than other states. Birth data correspond to the period between January 1, 2005, and December 31, 2016.

### Participants

We included all LB of mothers residing in São Paulo state, Brazil, whose information in the field of congenital malformations of the Live Birth Certificate was completed with Gastroschisis, identified by the following International Classification of Diseases, 10^th^ Revision, ICD–10: Q79.3. We excluded patients who had a main or secondary diagnosis of Gastroschisis and who had Omphalocele/Exonphalia (ICD–10: Q79.2), hypoplasia/malformation of the abdominal muscles (ICD–10: Q79.5), or umbilical hernia (ICD–10: K42).

### Variables

To describe the outcome of this study, sociodemographic and clinical explanatory variables were selected: gender, race/color, gestational age, birth weight, maternal age, maternal schooling, maternal occupation, type of gestation, type of delivery and the number of prenatal consultations.

The number of LB in São Paulo state is provided by SINASC. To construct the rates, they were stratified according to maternal age group and territorial clusters, year by year (for trend) and month by month (for seasonality).

**Figure 4:**
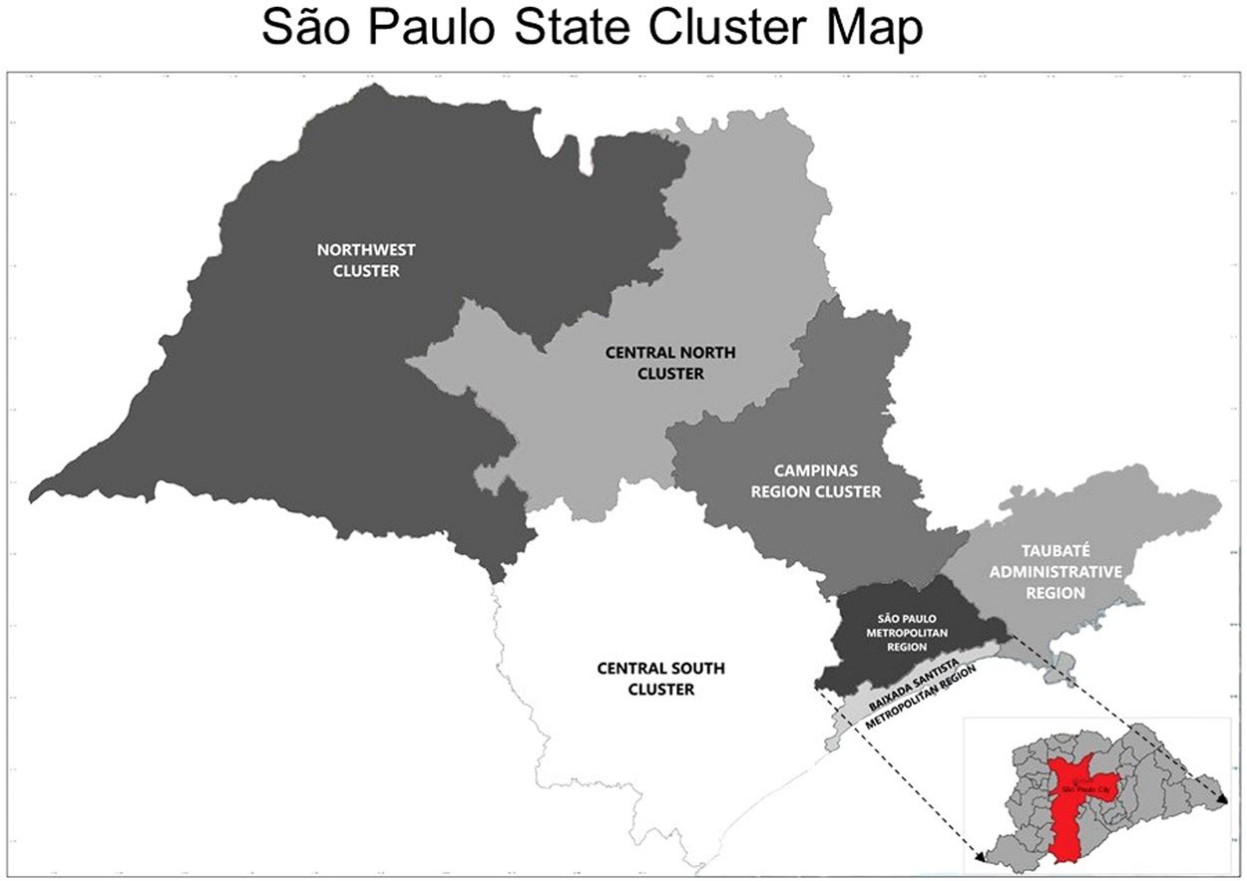
São Paulo State Cluster Map.

Gastroschisis trends of prevalence were calculated according to maternal age (≤14 years, 15–19 years, 20–24 years, 25–29 years, 30–34 years, ≥35 years) and for territorial clusters determined by the maternal address (São Paulo city – SPC, São Paulo metropolitan region without São Paulo city – SPMR without SPC, São Paulo metropolitan region – SPMR, Baixada Santista metropolitan region – BSMR, Taubaté Administrative region – TAR, Central South cluster – CSC (Sorocaba and Registro Administrative regions), Campinas region cluster – CRC (Campinas, Piracicaba and São João da Boa Vista Administrative regions), Central North cluster – CNC (Bauru, Araraquara, Ribeirão Preto e Franca Administrative regions), Northwest cluster – NWC (Marília, Presidente Prudente, Araçatuba, São José do Rio Preto and Barretos Administrative regions), and São Paulo state – SPS) in São Paulo state (Fig. 4). These clusters were grouped by similar territorial characteristics from the geographical delimitation of the administrative areas previously stipulated by government agencies^39^ and used by DATASUS. This procedure ensured a sufficient number of cases and stabilized the analyzis.

### Data sources

The microdata was extracted from the file transfer service provided by DATASUS. The TABNET and TABWIN programs were used to consult the data. Those tabs were developed to perform fast tabulations on.DBF files, then the files were exported to.XLS version; further, the variables selected for this study were classified in EXCEL^®^. In order to minimize possible discrepancies, the data were collected by two different researchers independently.

The Live Birth Information System was developed by DATASUS to gather epidemiological information on births reported throughout the country to subsidize interventions related to women’s and children’s health for all levels of the Unified Health System (SUS – Sistema Único de Saúde), such as actions of attention to the pregnant woman and to the newborn, as well as the monitoring of the evolution of the SINASC’s historical series, that allows the identification of intervention priorities and contribution to the effective improvement of this information system. Through the Internet, DATASUS and the Health Surveillance Service (SVS – Sistema de Vigilância em Saúde) provide the main information for tabulation on the SINASC’s Databases.

### Bias

Only the population of LB was used to obtain the prevalence rates and the proportion of maternal age range did not differ in the linear regression model by clusters in São Paulo state (p > 0.05). With this, the result obtained by the analysis can be used for comparison to other studies.

Some limitations have been identified, inherent in the recording of public data, in the data collection stages and, in the quality of the information where there were fields with missing data, until the transmission of the data to the information systems.

### Statistical methods

Gastroschisis prevalence rates were calculated for 10,000 LB by maternal age group and territorial clusters. For prevalence, in addition to the global period (2005–2016) 2 time intervals of 6 consecutive years each (2005–2010 and 2011–2016) were used.

For trends analysis, the Prais–Winsten regression model, following Antunes and Cardoso^31^ methodological indications, were used. The dependent variable was the logarithm of the rates, and the independent variable, and the years of the historical series. The Annual Percent Change (APC) of the rates was also calculated, as suggested by Antunes and Waldman (2002)^40^.

The data modelling process includes, transforming the standardized rates into a base 10 logarithmic function using the Durbin–Watson test to measure the existence of the first–order autocorrelation of the time series composed of the annual coefficients, as well as to verify that the correlation was compatible with the random regression residuals hypothesis. Annual rates of increase or decrease (APC), according to maternal age and geographic clusters, were then calculated, with the respective confidence intervals (95% CI). This procedure makes it possible to classify gastroschisis trend, as increasing, decreasing, or stationary. The trend was, considered to be stationary when the coefficient was not significantly different from zero (p > 0.05)^31^. To facilitate the visualization of trends, the third order centred moving averages technique was performed for trends and for seasonality^40,41^, without the outliers (Figs 1–3).

To model seasonality we used monthly measurements for LB with gastroschisis. For monthly measurements, calendar month was numbered sequentially (totalling 66 months during 2011–2016). In addition, to identify seasonal variations, gastroschisis monthly prevalence rates were calculated on the basis of the date of conception. Antunes and Waldman^40^ methodological indications were used for the seasonality hypothesis test. The seasonal variation was considered significant if one or more of the coefficients of the seasonal term (B3 and B5 for Seno and B2 and B4 for Cosseno) were statistically different from zero (p < 0.05)^31^. All statistical analyses, were performed using STATA 15.1 (CollegeStation, TX, 2018) conducted between February 2018, and August 2019.

## Data Availability

The microdata used for this study are administered by the Live Births Information System (SINASC – Sistema de Informação sobre Nascidos Vivos), using data from the Unified Health System Department of Informatics (DATASUS – Departamento de Informática do Sistema Único de Saúde), maintained by the Ministry of Health of Brazil. DATASUS provides open public access to these data for any purposes. www.datasus.saude.gov.br, http://datasus.saude.gov.br/informacoes-de-saude/tabnet/estatisticas-vitais.
